# Propofol-Induced Metabolic, Apoptotic, and Wnt–Frizzled-Related Transcript Changes in MIA PaCa-2 Cells: In Vitro and Bioinformatic Analyses

**DOI:** 10.3390/cimb48090950

**Published:** 2026-09-17

**Authors:** Irmak Fatoş Korkmaz, Tugba Elgun, Çiğdem Aktas, Sajjad Eslamkhah, Sevgi Kocyigit Sevinc, Asiye Gok Yurttas

**Affiliations:** 1 Department of Anaesthesia, Bahçelievler State Hospital, University of Health Science, Istanbul 34668, Türkiye; korkmazfatos1112@gmail.com; 2 Medical Biology, Faculty of Medicine, Biruni University, Istanbul 34015, Türkiye; telgun@biruni.edu.tr; 3 Biruni Advanced Technology Application and Research Center (BAMER), Biruni University, Istanbul 34015, Türkiye; mtaghizad@biruni.edu.tr; 4 Department of Genetics, Aziz Sancar Institute of Experimental Medicine, Istanbul University, Istanbul 34452, Türkiye; cigdemaktas485@gmail.com; 5 Program of Pathology Laboratory Techniques, Vocational School, Biruni University, Istanbul 34015, Türkiye; 6 Department of Biophysics, Faculty of Medicine, Kütahya Health Sciences University, Kutahya 43100, Türkiye; sevgi.kocyigitsevinc@ksbu.edu.tr; 7 Department of Medical Biochemistry, Faculty of Medicine, Istanbul Atlas University, Istanbul 34403, Türkiye

**Keywords:** propofol, MIA PaCa-2, pancreatic ductal adenocarcinoma, Wnt–Frizzled, RT-qPCR, apoptosis

## Abstract

Pancreatic ductal adenocarcinoma (PDAC) remains a highly lethal malignancy, and there is continuing interest in whether agents used during the perioperative period influence tumour-cell biology. Propofol has been associated with antiproliferative and pro-apoptotic effects in experimental cancer models; however, its relationship with Wnt–Frizzled-related transcriptional responses in pancreatic cancer cells remains incompletely characterised. MIA PaCa-2 cells were exposed to propofol for 24 h. Cellular metabolic activity was assessed using the MTT assay over a concentration range of 0–20 µM, and apoptosis was evaluated via Annexin V-FITC/7-AAD flow cytometry at the MTT-derived IC_50_ concentration of 7.6 µM. Transcript abundance of 18 pathway-associated genes was analysed via RT-qPCR following exposure to 7.6 µM propofol. MTT, flow-cytometry, and RT-qPCR assays were each performed in three independent biological experiments (*n* = 3). RT-qPCR results were evaluated after Benjamini–Hochberg false discovery rate correction across the 18-transcript panel. Complementary bioinformatic analyses were performed solely to provide exploratory biological and clinical context for the experimentally prioritised transcripts. Propofol produced a concentration-dependent reduction in normalised MTT metabolic activity, with an MTT-derived IC_50_ of 7.6 µM. At this concentration, total apoptosis increased from 0.67 ± 0.11% in vehicle-treated cells to 17.04 ± 2.40% following propofol exposure (*p* < 0.001), whereas necrosis remained minimal. The viable-cell fraction decreased from 99.30 ± 1.057% to 82.80 ± 0.841% (*p* < 0.05). The difference between the MTT-derived IC_50_ and the Annexin V/7-AAD viable-cell fraction indicates that the MTT-derived IC_50_ reflects reduced metabolic activity rather than 50% cell lethality. RT-qPCR showed decreased WNT5B, FZD5, and WNT11 transcript abundance and increased GSK3B transcript abundance. All four remained significant after Benjamini–Hochberg correction (WNT5B, q = 0.001; FZD5, q = 0.001; WNT11, q = 0.027; GSK3B, q = 0.041). Exploratory bioinformatic analyses showed heterogeneous gene-specific expression, survival, genomic, immune-infiltration, and dependency patterns and were not interpreted as evidence of a propofol-mediated mechanism in patients. Under the tested in vitro conditions, propofol reduced MTT-derived metabolic activity, increased apoptosis, and was associated with selective Wnt–Frizzled-related transcript changes in MIA PaCa-2 cells. These transcript-level observations do not establish direct functional modulation of Wnt–Frizzled signalling or demonstrate that this pathway mediates the observed apoptotic response. The bioinformatic findings provide exploratory contextual information only. Further validation in additional PDAC and non-malignant pancreatic cell models, together with protein-, phosphorylation-, and pathway-level functional studies, is required.

## 1. Introduction

Pancreatic ductal adenocarcinoma (PDAC) remains among the most lethal solid malignancies, owing to early metastatic dissemination, limited therapeutic responsiveness, and frequent recurrence despite advances in surgery and systemic treatment [1,2,3]. Because potentially resectable disease is managed surgically, the perioperative period has attracted interest as a biological window during which anaesthetic and analgesic exposures may influence tumour-cell behaviour. This possibility is biologically plausible, but clinical evidence remains unsettled and should not be inferred directly from experimental cell-culture studies.

Propofol is a fast-acting intravenous anaesthetic widely used for the induction and maintenance of general anaesthesia. In experimental cancer models, propofol has been reported to reduce proliferation, promote apoptosis, and alter migration or invasion, including in pancreatic cancer cells [4,5,6,7,8,9]. Proposed mechanisms include effects on NF-κB, PI3K/AKT, and other signalling networks, although the direction and magnitude of these effects vary by tumour model, exposure concentration, and assay system [6,8,9].

Wnt–Frizzled signalling is relevant to PDAC because both canonical and non-canonical branches contribute to tumour growth, plasticity, stemness, and invasive behaviour [5,10,11]. Propofol has been associated with Wnt-related molecular changes in other tumour models [12], but direct pathway activity cannot be inferred from transcript abundance alone. We therefore asked a narrower question: whether 24 h propofol exposure is associated with changes in selected Wnt–Frizzled-related transcripts in MIA PaCa-2 cells, alongside changes in MTT metabolic activity and apoptotic phenotype. Complementary public-dataset analyses were used solely to contextualise the four transcripts identified in vitro; they were not intended as evidence that propofol alters patient tumours, clinical outcomes, or directly modulates Wnt–Frizzled signalling.

## 2. Materials and Methods

### 2.1. Cell Culture

MIA PaCa-2 human pancreatic carcinoma cells were obtained from the American Type Culture Collection (ATCC, Manassas, VA, USA) and maintained in RPMI-1640 medium (Gibco, Life Technologies Corporation, Grand Island, NY, USA) supplemented with 10% fetal bovine serum and 1% penicillin–streptomycin at 37 °C in a humidified atmosphere containing 5% CO_2_.

### 2.2. Propofol Treatment

Propofol (Sigma-Aldrich, St. Louis, MO, USA; ≥97% purity) was dissolved in dimethyl sulfoxide (DMSO) and diluted in culture medium immediately before use to final concentrations of 1, 5, 10, and 20 µM. The final DMSO concentration did not exceed 0.1% in any condition, and vehicle-control cells received the corresponding volume of DMSO. Exposure time was 24 h.

### 2.3. MTT Metabolic Activity Assay

Cells were seeded in 96-well plates at 1 × 10^4^ cells/well and allowed to attach overnight. After 24 h of propofol exposure, 100 µL of MTT solution (0.4 mg/mL) was added and the plates were incubated for 3 h at 37 °C. Formazan was solubilised in 100 µL of DMSO and absorbance was measured at 550 nm. The MTT signal was normalised to the vehicle-control group and expressed as relative metabolic activity (%). Concentration–response data were fitted to a four-parameter logistic model to estimate the concentration producing a 50% reduction in the MTT signal (MTT-derived IC_50_). This value was not interpreted as a concentration causing 50% cell death.

### 2.4. Apoptosis Analysis

Apoptosis and necrosis were assessed after 24 h of exposure to 7.6 µM propofol, with the concentration corresponding to the MTT-derived IC_50_, using an Annexin V-FITC/7-AAD kit (BioLegend, San Diego, CA, USA). Cells were seeded at 6 × 10^5^ cells/well in 6-well plates. After 24 h of treatment, both non-adherent/floating cells present in the culture supernatant and adherent cells were collected and pooled to ensure inclusion of detached apoptotic cells. The combined cell population was washed with PBS, resuspended in binding buffer, and stained with Annexin V-FITC and 7-AAD according to the manufacturer’s protocol. Acquisition was performed on a BD Accuri C6 Plus flow cytometer (BD Biosciences, Franklin Lakes, NJ, USA), with at least 10,000 events acquired per sample. Vehicle-treated cells were analysed in parallel. Quadrant gates were established using unstained and single-stained controls. Viable cells were defined as Annexin V−/7-AAD−, early apoptotic cells as Annexin V+/7-AAD−, late apoptotic cells as Annexin V+/7-AAD+, and necrotic cells as Annexin V−/7-AAD+. Total apoptosis was calculated as the sum of the early and late apoptotic populations.

### 2.5. RNA Isolation and RT-qPCR

Cells used for RT-qPCR analysis were treated with 7.6 µM propofol, corresponding to the experimentally determined MTT-derived IC_50_, for 24 h. Total RNA was isolated using the innuPREP RNA Mini Kit 2.0 (Analytik Jena AG, Jena, Germany). One microgram of RNA was reverse transcribed using the OneScript Plus cDNA Synthesis Kit (Applied Biological Materials Inc. [abm], Richmond, BC, Canada). RT-qPCR was performed on a Roche LightCycler 480 using the GeneQuery Human Basal Cell Carcinoma qPCR Array (ScienCell Research Laboratories, GQH-BCC-GK015-C, Carlsbad, CA, USA). The array was used as a targeted panel because it contains validated primer sets covering Wnt–Frizzled ligands, receptors, and intracellular pathway-associated genes; it was not used as a basal cell carcinoma classifier. Each 20 µL reaction contained 10 µL of 2× GoldNStart TaqGreen qPCR Master Mix (Cat. No. MB6018; ScienCell Research Laboratories, Carlsbad, CA, USA), 500 ng of cDNA, and RNase-free water. The cycling conditions were 95 °C for 10 min, followed by 40 cycles of 95 °C for 20 s, 65 °C for 20 s, and 72 °C for 20 s, followed by melt-curve analysis to confirm single-product amplification. RT-qPCR experiments were performed in three independent biological experiments (*n* = 3), with each sample analysed in technical duplicate. Technical duplicate Cq values were averaged before statistical analysis. GAPDH was used as the reference gene, and relative transcript abundance was calculated using the 2^−ΔΔCq^ method.

### 2.6. Exploratory Bioinformatic Contextualisation

To provide broader biological and clinical context for the transcripts prioritised from the in vitro RT-qPCR analysis, complementary bioinformatic analyses were performed using publicly available cancer datasets. These analyses were restricted primarily to GSK3B, WNT5B, WNT11, and FZD5 and were designed solely for exploratory contextualisation rather than mechanistic validation. Tumour-versus-normal and stage-stratified transcript-expression patterns were examined using UALCAN and GEPIA2 based on TCGA-PAAD data. Genomic alterations, including somatic mutations and copy-number alterations, together with copy-number–expression relationships, were explored using cBioPortal and GISTIC-derived copy-number states. Tumour-purity-adjusted associations between gene expression and estimated immune-cell infiltration were evaluated using TIMER 2.0 with partial Spearman correlation analyses. Functional dependency was explored using DepMap 24Q2 CRISPR–CERES gene-effect data. Overall survival was evaluated using the KM-Plotter pancreatic-cancer dataset with the platform’s optimised-cutoff option, and hazard ratios (HRs), 95% confidence intervals (CIs), and log-rank *p* values were recorded. Exploratory KEGG pathway-enrichment analysis and DGIdb drug–gene interaction mapping were additionally used to provide functional and pharmacological context for the analysed gene set.

Importantly, none of these computational analyses directly modelled propofol exposure. The bioinformatic findings were therefore interpreted exclusively as exploratory and hypothesis-generating contextual information and were not used to establish causality, independently validate a propofol-mediated molecular mechanism, or infer equivalent molecular effects in patient tumours.

### 2.7. Statistical Analysis

Statistical analyses were performed using GraphPad Prism 10.6.1 (GraphPad Software, San Diego, CA, USA). The MTT, flow cytometry, and RT-qPCR experiments were each performed as three independent biological experiments (*n* = 3), conducted separately for each assay. Independent biological replicates were defined as experiments initiated from separate cell passages and performed on different days. Technical replicates within each independent experiment were averaged before statistical analysis and were not treated as independent observations. Data are presented as the mean ± standard deviation (SD), with individual biological replicate values displayed where applicable. All statistical tests were two-tailed, and *p* < 0.05 was considered statistically significant.

For the MTT concentration–response assay, vehicle-treated and propofol-treated groups were compared using one-way analysis of variance (ANOVA) followed by Dunnett’s multiple-comparisons test, with each propofol concentration compared with the vehicle control. Homogeneity of variance was assessed using the Brown–Forsythe test. When the assumption of equal variances was not satisfied, Brown–Forsythe and Welch ANOVA followed by Dunnett’s T3 multiple-comparisons test were applied. The MTT-derived half-maximal inhibitory concentration (IC_50_) and its 95% confidence interval (CI) were estimated by nonlinear regression using a four-parameter variable-slope concentration–response model.

For flow-cytometry analysis, the percentages of viable, early apoptotic, late apoptotic, necrotic, and total apoptotic cells were calculated for each independent biological experiment. Proportion data were arcsine square-root transformed before inferential statistical analysis, whereas untransformed percentages are presented in the figures and tables for interpretability. Vehicle-treated and 7.6 µM propofol-treated groups were compared using the corresponding replicate-level statistical analysis, and exact *p* values are reported for each cell population.

RT-qPCR statistical inference was performed using ΔCq values rather than fold-change values. Technical duplicate Cq measurements were averaged for each biological replicate before analysis. For each of the 18 genes, ΔCq values from vehicle-treated and 7.6 µM propofol-treated cells were compared, and the resulting raw *p* values were adjusted for multiple testing using the Benjamini–Hochberg false discovery rate procedure (Q = 5%) across the complete 18-gene family. Both raw *p* values and Benjamini–Hochberg-adjusted q values are reported. Statistical significance after multiple-testing correction was defined as q < 0.05.

## 3. Results

### 3.1. Propofol Produces a Concentration-Dependent Reduction in MTT Metabolic Activity

**Figure 1:**
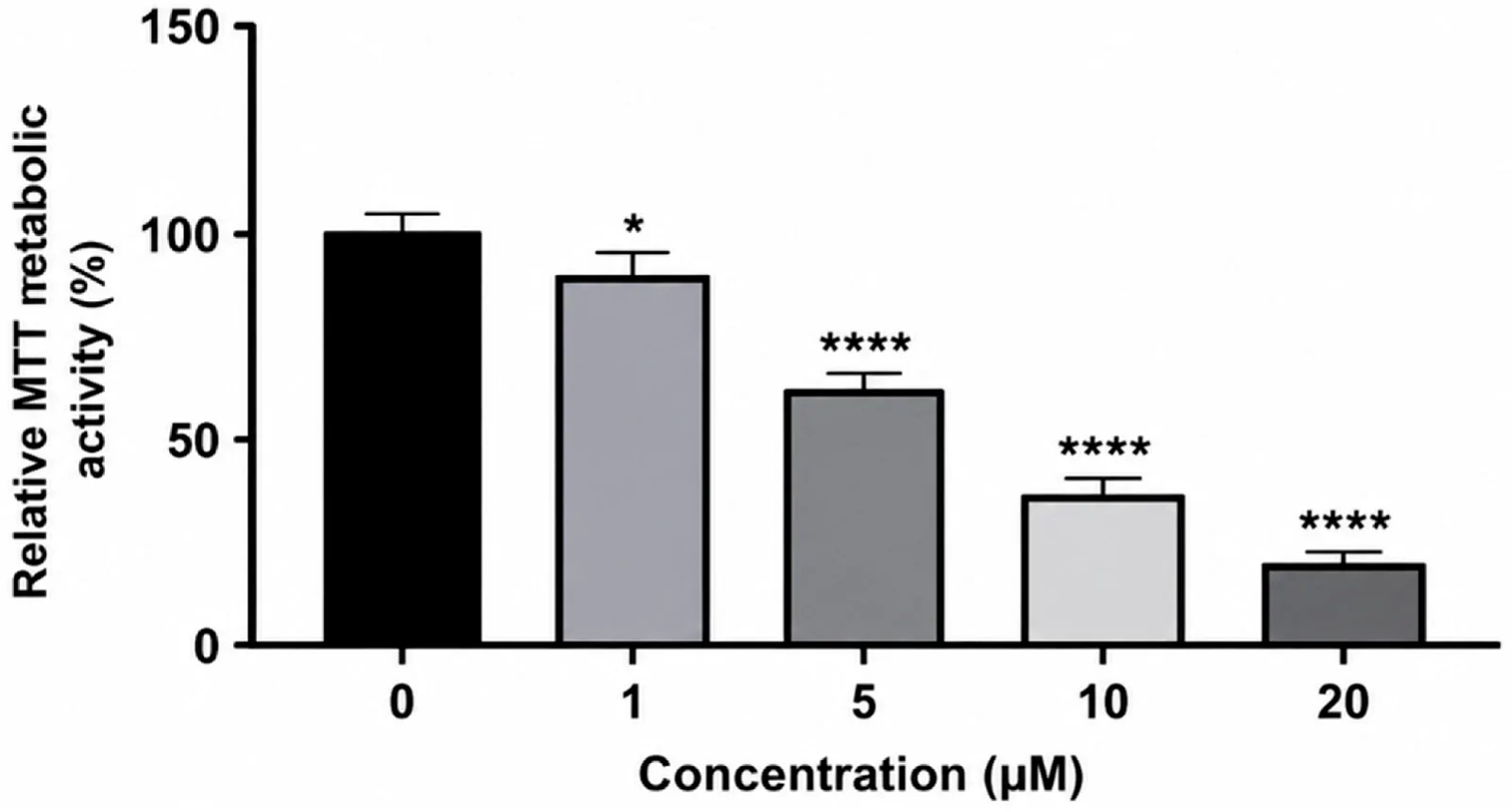
Relative MTT metabolic activity of MIA PaCa-2 cells following 24 h of exposure to propofol. Cells were treated with 0, 1, 5, 10, or 20 µM propofol. MTT activity was normalised to the vehicle control and expressed as a percentage of control. Data are presented as mean ± SD from three independent biological experiments (*n* = 3). Statistical comparisons with the vehicle control were performed using one-way ANOVA followed by Dunnett’s multiple-comparisons test. * *p* < 0.05; **** *p* < 0.0001. The MTT-derived IC_50_ was 7.6 µM.

Propofol treatment produced a concentration-dependent reduction in the normalised MTT signal after 24 h of exposure (Figure 1). Relative metabolic activity was 100% in the vehicle-treated control group and decreased to 89.9%, 62.3%, 33.6%, and 16.1% following treatment with 1, 5, 10, and 20 µM propofol, respectively. Nonlinear concentration–response analysis yielded an MTT-derived IC_50_ of 7.6 µM. Because the MTT assay reflects cellular tetrazolium-reducing metabolic activity rather than directly measuring cell number or membrane-defined cell death, this IC_50_ represents a 50% reduction in the normalised MTT signal relative to the vehicle control and should not be interpreted as indicating 50% cell lethality.

### 3.2. Propofol Is Associated with an Increased Apoptotic Fraction at the MTT-Derived IC_50_

**Figure 2:**
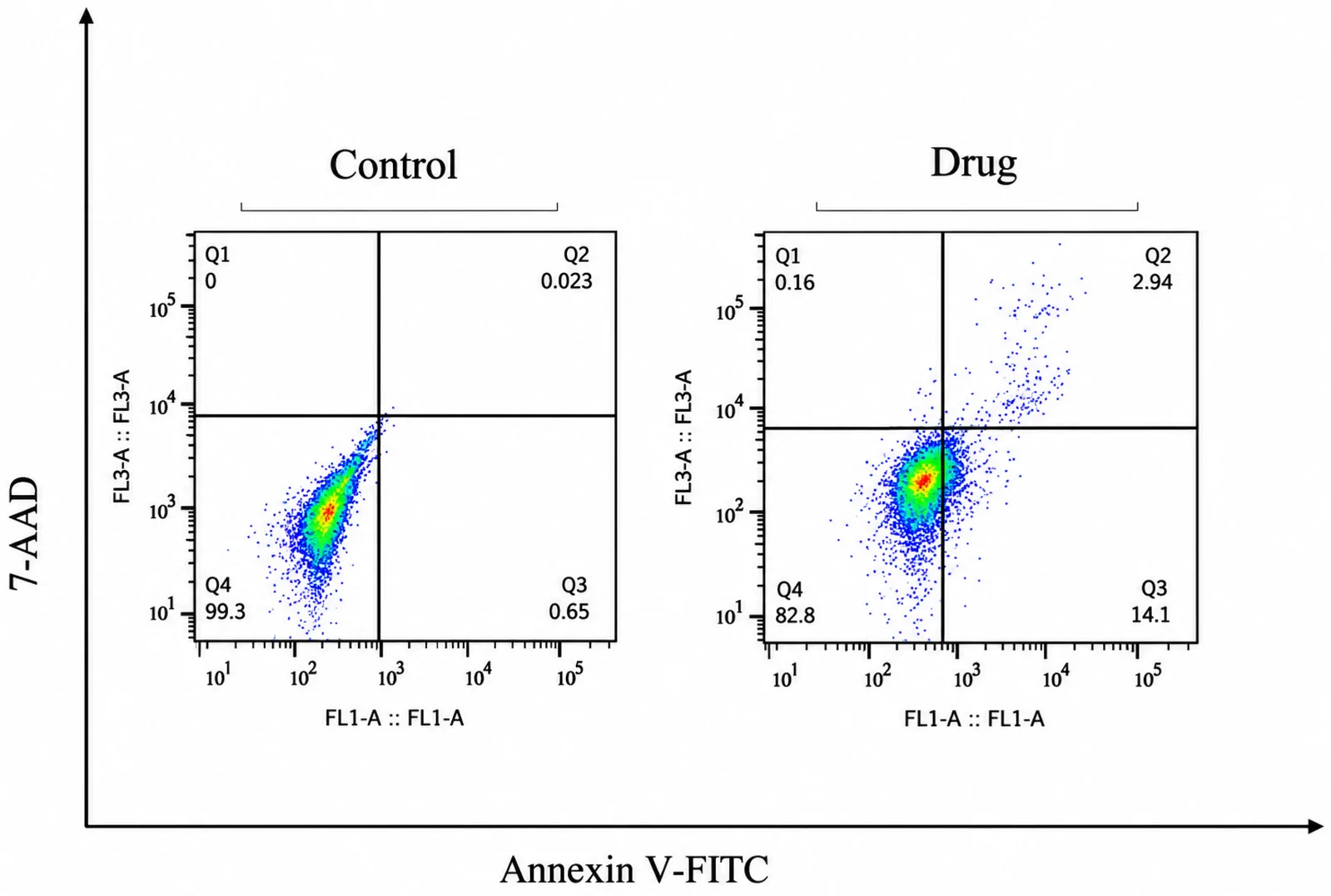
Representative Annexin V-FITC/7-AAD flow-cytometry plots of MIA PaCa-2 cells after 24 h of exposure to vehicle or 7.6 µM propofol. Viable cells: Annexin V−/7-AAD−; early apoptosis: Annexin V+/7-AAD−; late apoptosis: Annexin V+/7-AAD+; necrosis: Annexin V−/7-AAD+. The displayed percentages are from the representative experiment; quantitative inference should be based on the independent biological experiments summarised in Table 1.

Annexin V-FITC/7-AAD flow cytometry was used to characterise the cell-death profile of MIA PaCa-2 cells following 24 h of exposure to 7.6 µM propofol. Re-examination of the replicate-level flow-cytometry data confirmed that the previously reported standard deviations for the viable-cell fractions contained decimal-place transcription errors. The corrected values were 99.30 ± 1.057% for vehicle-treated cells and 82.80 ± 0.841% for propofol-treated cells. Early apoptotic cells increased from 0.65% to 14.1%, while late apoptotic cells increased from 0.02% to 2.94%. Necrotic cells remained minimal, accounting for 0.16% of the propofol-treated population. Accordingly, the total apoptotic fraction in the representative experiment increased from 0.67% in the vehicle control to 17.04% following propofol exposure. Figure 2 presents representative flow-cytometry plots, whereas quantitative comparisons and statistical analyses were based on three independent biological experiments (*n* = 3) and are summarised as the mean ± SD in Table 1.

### 3.3. Propofol Exposure Is Associated with Selective Wnt–Frizzled-Related Transcript Changes

Among the 18 transcripts analysed, the most pronounced expression changes following 24 h of exposure to 7.6 µM propofol were observed for WNT5B, FZD5, WNT11, and GSK3B. Relative to the vehicle-treated control, WNT5B, FZD5, and WNT11 transcript levels were decreased, with fold changes of 0.25, 0.36, and 0.42, respectively, whereas GSK3B transcript abundance increased 2.10-fold. The remaining transcripts showed smaller expression changes and did not meet the prespecified combined fold-change and statistical criteria in the initial analysis. Because 18 genes were evaluated in parallel, statistical significance was assessed after correction for multiple testing using the Benjamini–Hochberg false discovery rate procedure (Q = 5%). Accordingly, raw *p* values and Benjamini–Hochberg-adjusted q values are presented in Table 2, and statistical significance after correction was defined as q < 0.05. Fold-change values are presented to indicate the magnitude and direction of the observed transcriptional changes and are not interpreted independently as evidence of statistical significance. After Benjamini–Hochberg correction across the complete 18-transcript family, all four transcripts identified in the initial analysis remained statistically significant: WNT5B (q = 0.001), FZD5 (q = 0.001), WNT11 (q = 0.027), and GSK3B (q = 0.041). The remaining 14 transcripts did not reach statistical significance after FDR correction (all q > 0.05).

### 3.4. Exploratory Expression and Survival Contextualisation in PDAC

**Figure 3:**
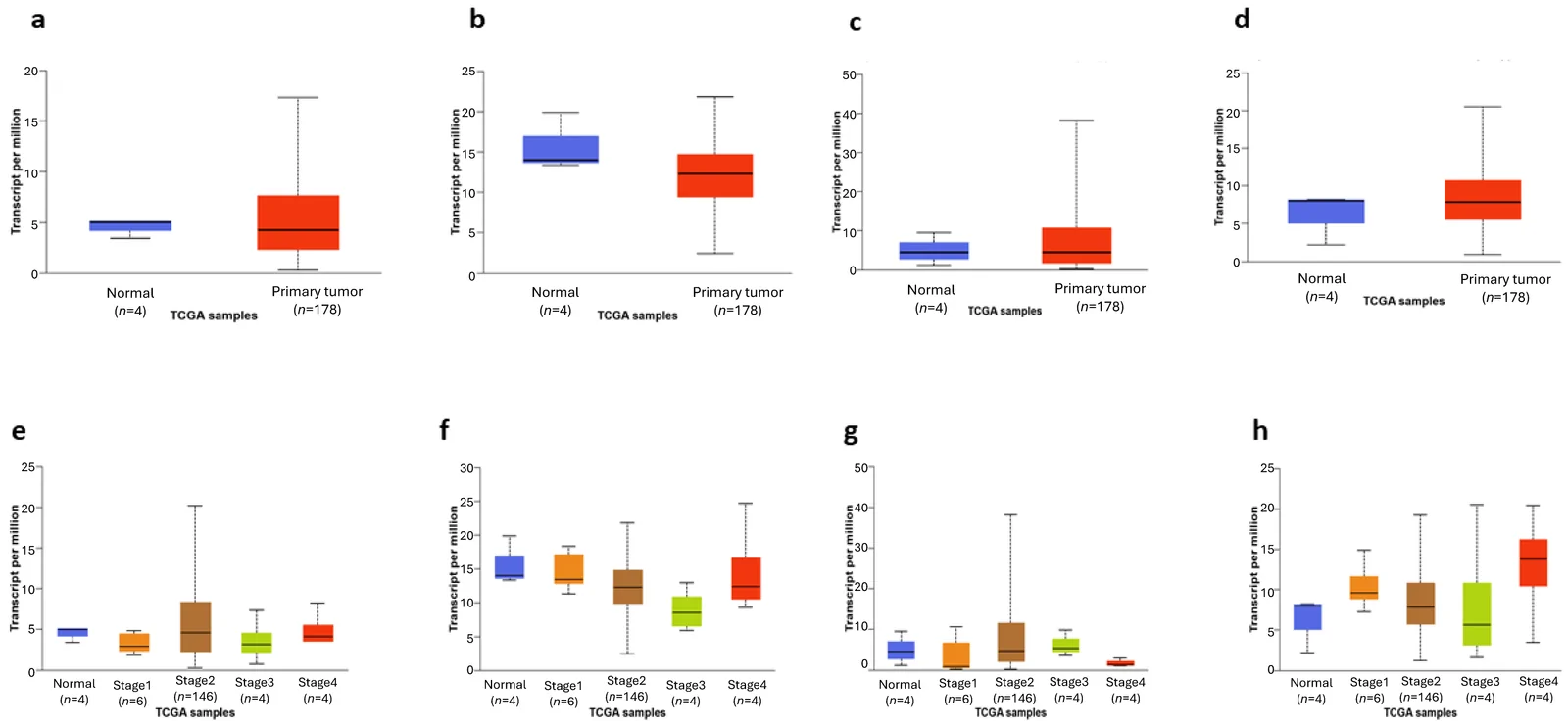
Exploratory tumour-versus-normal (**a**–**d**) and stage-stratified expression (**e**–**h**) of GSK3B, WNT5B, WNT11, and FZD5 in the TCGA-PAAD dataset. These analyses provide descriptive clinical context and should not be interpreted as evidence of a propofol-mediated effect in patients.

To provide exploratory clinical context for the four transcripts prioritised from the in vitro analysis, GSK3B, WNT5B, WNT11, and FZD5 were examined in publicly available pancreatic-cancer datasets. All four genes showed higher transcript abundance in pancreatic tumour tissue than in normal pancreatic tissue. Stage-stratified analyses additionally demonstrated variation in expression across clinical stages; however, these patterns were considered descriptive and were not interpreted as evidence of progressive pathway activation, a propofol-mediated effect in patients, or a causal relationship with the in vitro findings (Figure 3a–h).

**Figure 4:**
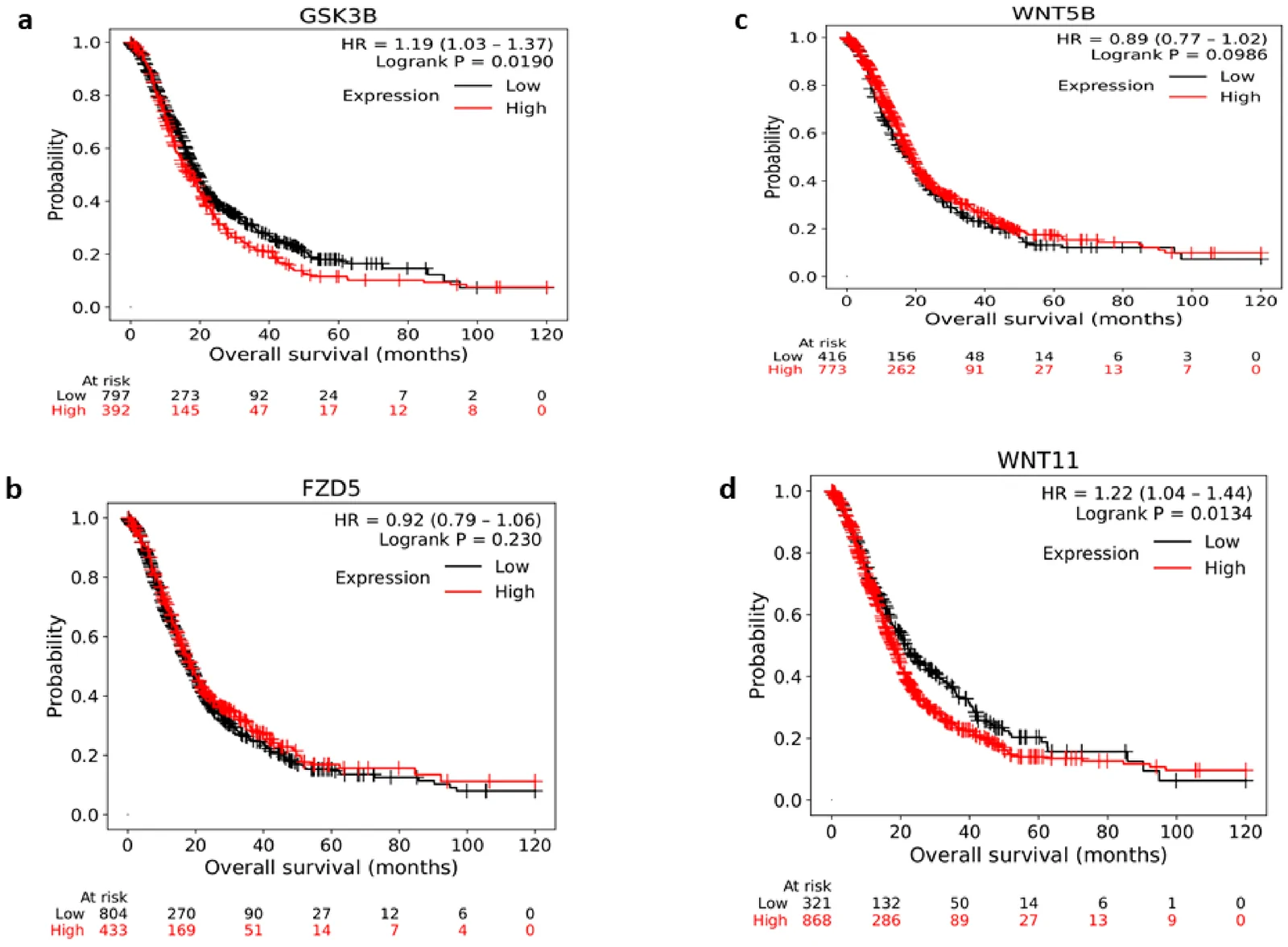
Overall survival according to (**a**) GSK3B, (**b**) FZD5, (**c**) WNT5B, and (**d**) WNT11 expression in the pancreatic-cancer dataset. Kaplan–Meier curves were generated using the KM-Plotter pancreatic-cancer dataset and its optimised-cutoff option. (**a**) GSK3B: HR = 1.19, 95% CI 1.03–1.37, *p* = 0.0190. (**b**) FZD5: HR = 0.92, 95% CI 0.79–1.06, *p* = 0.230. (**c**) WNT5B: HR = 0.89, 95% CI 0.77–1.02, *p* = 0.0986. (**d**) WNT11: HR = 1.22, 95% CI 1.04–1.44, *p* = 0.0134. This analysis provides exploratory gene-level clinical context and is not evidence of a clinical effect of propofol.

Survival analysis revealed gene-specific associations. Higher GSK3B expression was associated with shorter overall survival (HR = 1.19, 95% CI 1.03–1.37; log-rank *p* = 0.019), as was higher WNT11 expression (HR = 1.22, 95% CI 1.04–1.44; *p* = 0.013). FZD5 expression was not significantly associated with overall survival (HR = 0.92, 95% CI 0.79–1.06; *p* = 0.230), whereas higher WNT5B expression showed a non-significant trend toward longer survival (HR = 0.89, 95% CI 0.77–1.02; *p* = 0.099). These survival associations were examined solely to contextualise the clinical behaviour of the selected transcripts and do not indicate that propofol influences patient survival or tumour behaviour (Figure 4a–d).

The direction of the in vitro transcript changes was not uniformly concordant with the patient survival associations. This discordance further supports cautious interpretation and indicates that propofol-associated transcript changes observed in MIA PaCa-2 cells cannot be directly translated into prognostic or functional effects in patients.

### 3.5. Exploratory Genomic Context of the Prioritised Transcripts

**Figure 5:**
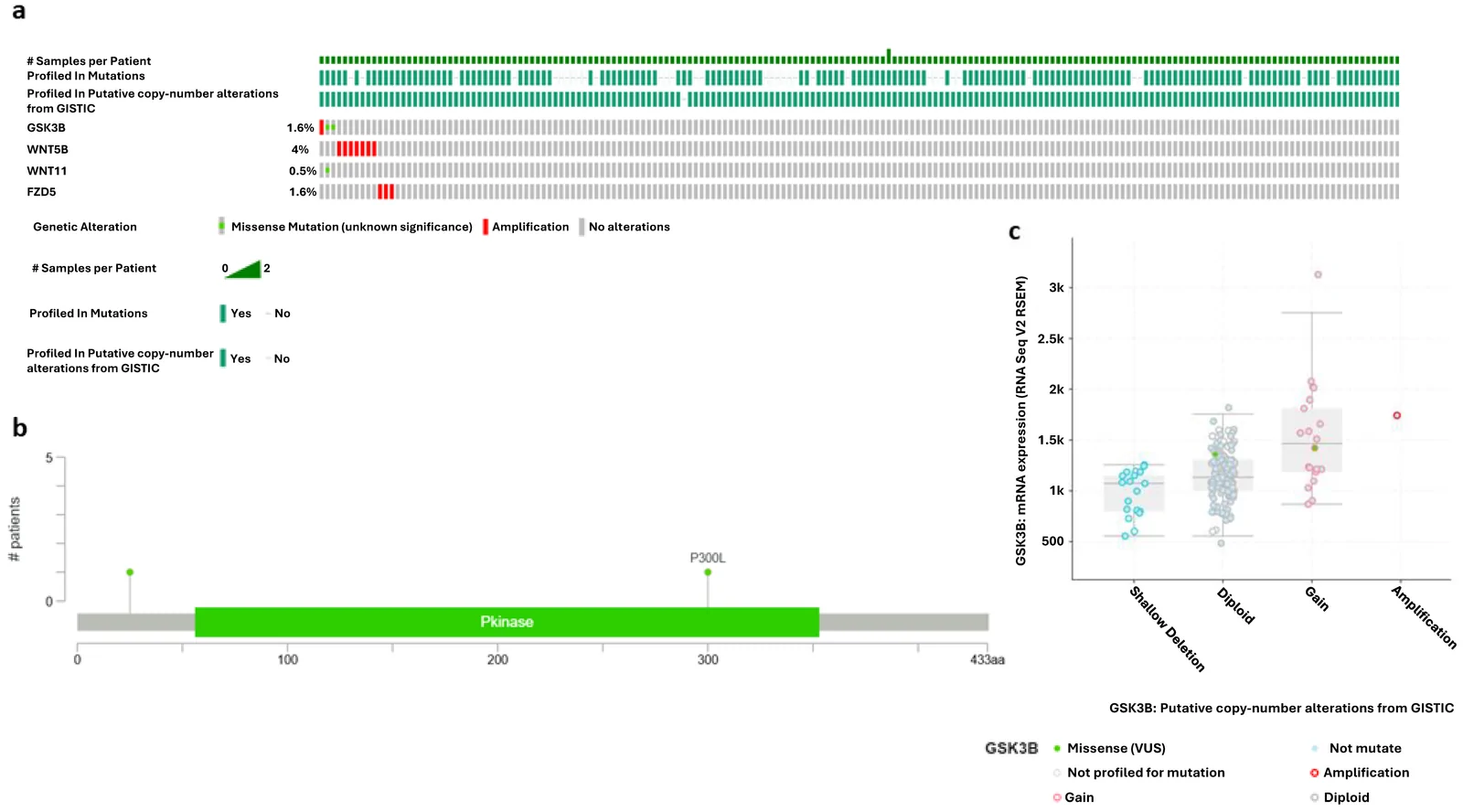
Exploratory genomic alterations (**a**,**b**) and copy-number–expression relationships (**c**) of GSK3B, WNT5B, WNT11, and FZD5 in pancreatic cancer. These findings provide genomic context only and do not establish a relationship with propofol exposure.

Genomic alterations were relatively infrequent across GSK3B, WNT5B, WNT11, and FZD5. WNT5B showed the highest alteration frequency, predominantly involving focal amplification, whereas GSK3B and FZD5 exhibited shallow copy-number gains with occasional missense mutations; WNT11 remained largely genomically stable. GSK3B lollipop mapping demonstrated sparse non-synonymous mutations without an evident recurrent hotspot. Integration of GISTIC-derived copy-number states with transcript abundance showed higher mRNA levels in tumours with copy-number gain or amplification, indicating an association between copy-number status and gene expression (Figure 5a–c). These findings provide exploratory genomic context for the selected genes but do not establish a relationship with propofol exposure.

### 3.6. Exploratory Immune-Infiltration Context of GSK3B Expression

**Figure 6:**
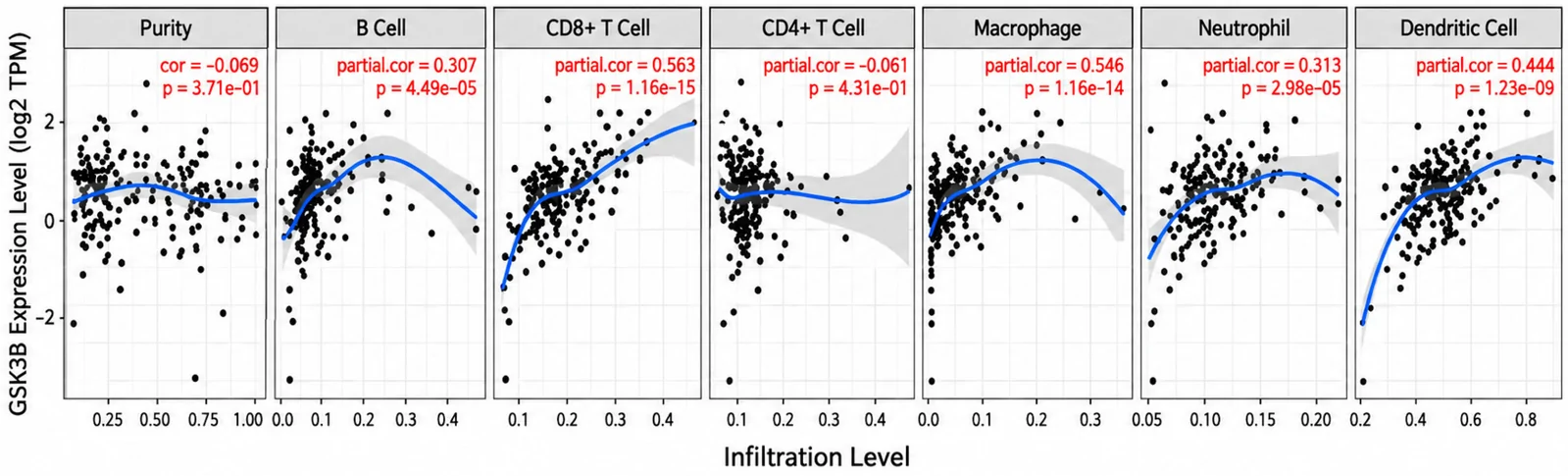
Exploratory tumour-purity-adjusted correlations between GSK3B expression and estimated immune-cell infiltration in pancreatic cancer. These computational associations do not establish immune-cell recruitment by GSK3B or an effect of propofol on tumour immune infiltration.

Tumour-purity-adjusted partial Spearman correlation analysis showed positive associations between GSK3B expression and estimated infiltration of CD8^+^ T cells, macrophages, dendritic cells, neutrophils, and B cells, whereas the association with CD4^+^ T-cell infiltration was weaker. These findings provide exploratory immune-contextual information for GSK3B expression in pancreatic cancer but do not establish a causal relationship between GSK3B expression, immune-cell recruitment, or propofol exposure (Figure 6).

### 3.7. Exploratory DepMap Dependency Context

**Figure 7:**
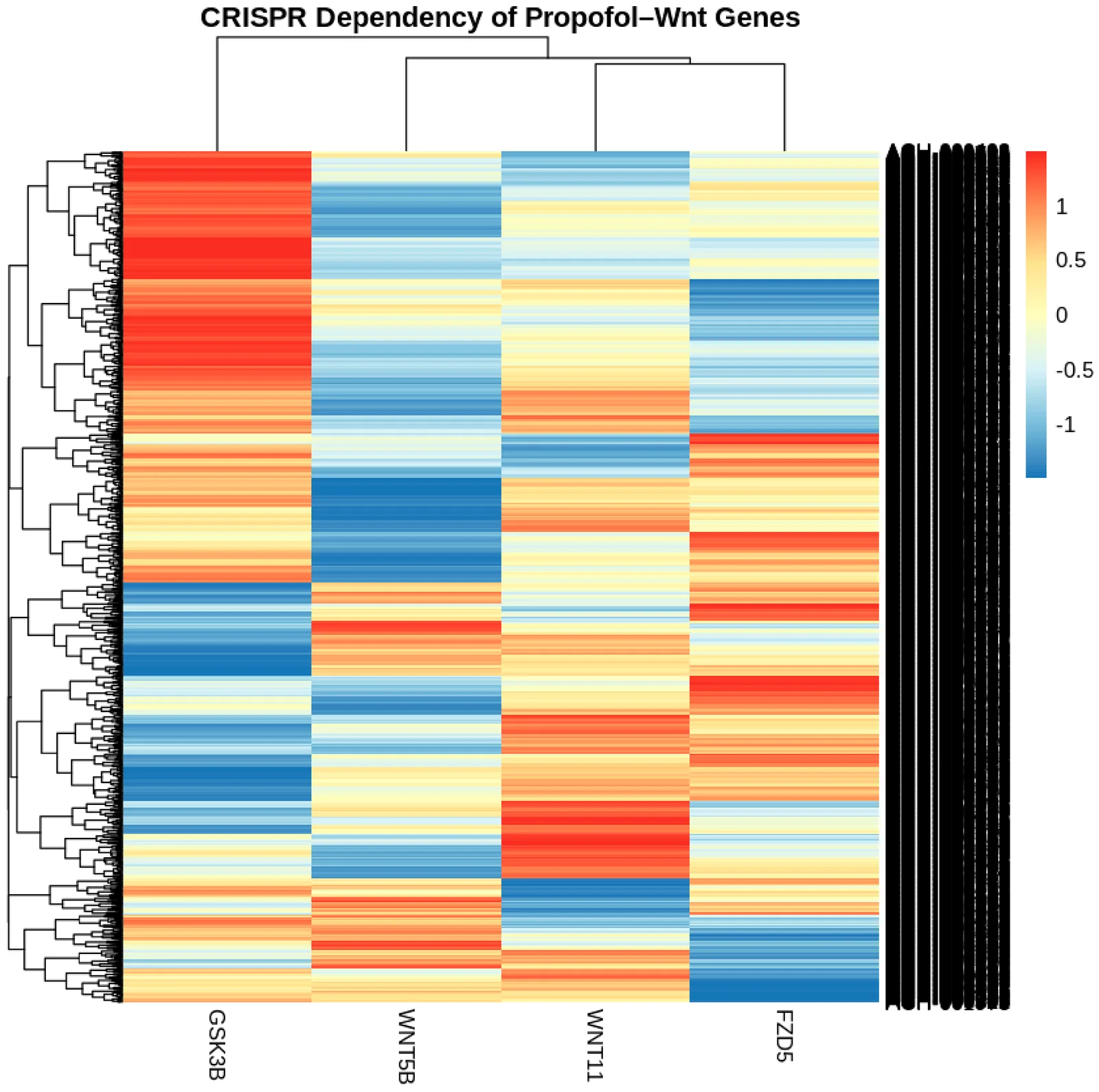
Exploratory CRISPR–CERES gene-effect profiles of GSK3B, WNT5B, WNT11, and FZD5 across cancer cell lines in the DepMap dataset. More negative gene-effect scores indicate greater dependence on the corresponding gene for cellular fitness. GSK3B showed a comparatively stronger negative dependency pattern than WNT5B, WNT11, and FZD5; however, its values did not consistently indicate strong or common-essential dependency. This analysis provides functional contextualisation only and does not demonstrate a causal relationship with propofol exposure.

In DepMap 24Q2, GSK3B showed a comparatively stronger negative gene-effect pattern than WNT5B, WNT11, and FZD5. However, the observed values did not consistently reach the range typically associated with strong or common-essential dependency. WNT5B, WNT11, and FZD5 showed weaker and more heterogeneous dependency profiles across cancer cell lines. These findings are presented solely as exploratory functional context and should not be interpreted as evidence of broad GSK3B essentiality or as proof that propofol-associated transcript changes directly determine cellular dependency or viability (Figure 7).

### 3.8. Exploratory Drug–Gene Interaction Mapping

Exploratory interrogation of DGIdb identified previously reported drug–gene associations involving selected genes represented in the analysed pathway network. These included GSK3B–lithium, FZD5–vantictumab, WNT11–LGK974, APC–aspirin, and CSNK1G2–PF-670462 associations (Table 3). Importantly, APC and CSNK1G2 were included only as pathway-context genes and were not among the four transcripts showing the most pronounced propofol-associated expression changes in the RT-qPCR experiment. Accordingly, these interactions are presented solely as pharmacological contextualisation of the broader gene network and should not be interpreted as evidence that propofol acts through these drug targets, that the listed agents would reproduce or enhance the effects of propofol, or that any therapeutic combination is supported by the present data.

### 3.9. Exploratory KEGG Pathway Enrichment Provides Functional Context for the Analysed Gene Set

**Figure 8:**
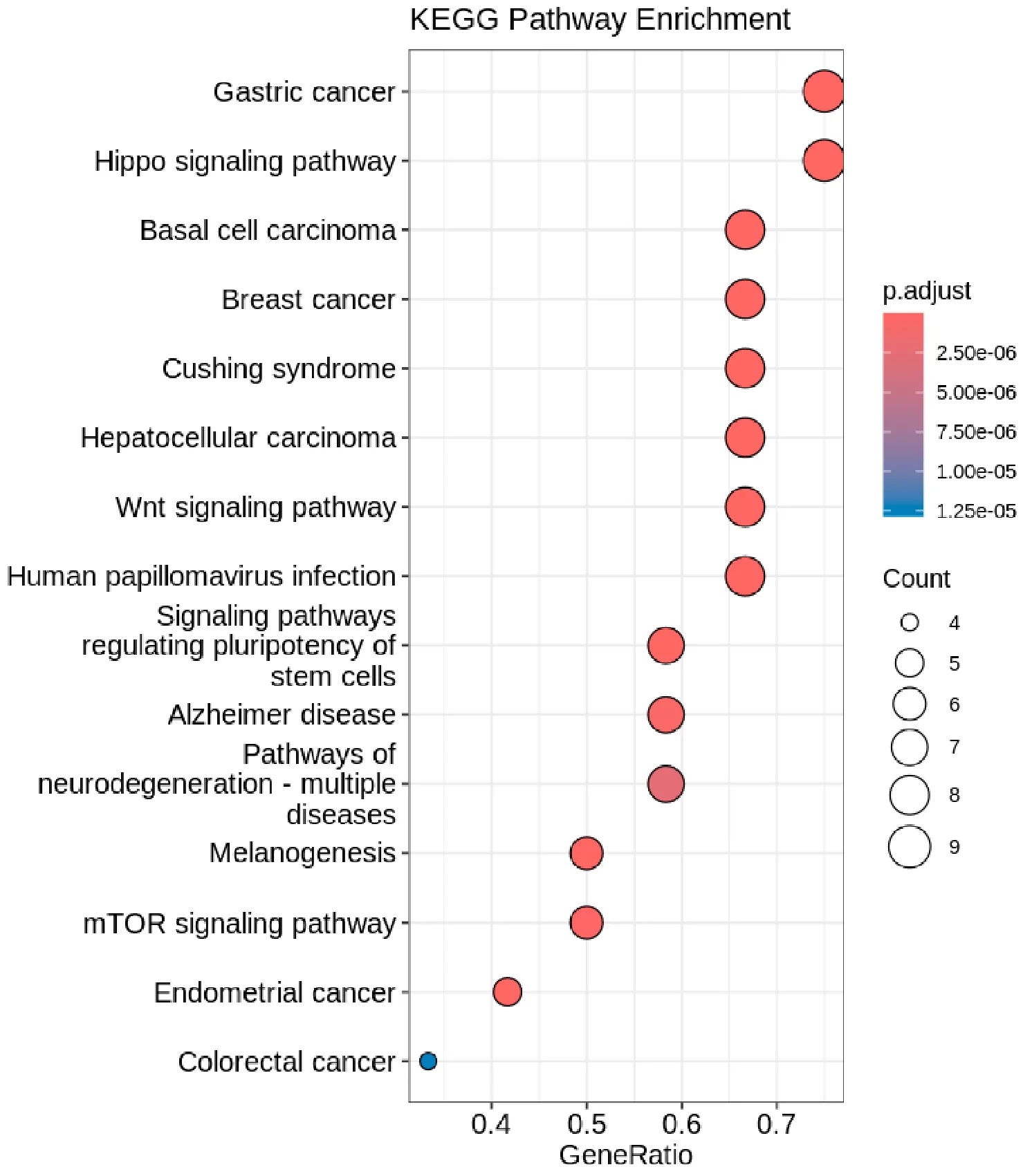
Exploratory KEGG pathway enrichment analysis of the gene set included in the bioinformatic analysis. Bubble position represents the gene ratio for each enriched pathway, bubble size indicates the number of contributing genes, and colour represents the adjusted *p* value. Cancer- and signalling-related pathways, including the Wnt signalling pathway, were represented among the enriched terms. Because the analysis was based on a targeted gene panel, the results are presented as functional contextualisation and should not be interpreted as independent evidence of pathway-level modulation by propofol.

Exploratory KEGG pathway enrichment analysis identified enrichment of several cancer- and signalling-related pathways, including gastric cancer, Hippo signalling, basal cell carcinoma, breast cancer, Cushing syndrome, hepatocellular carcinoma, and the Wnt signalling pathway. Because the input genes were derived from a targeted carcinoma-associated RT-qPCR panel rather than from unbiased transcriptome-wide profiling, these enrichment results should be interpreted as functional contextualisation of the analysed gene set rather than as independent evidence that propofol activates or suppresses these pathways. The presence of Wnt-related and cancer-associated pathways among the enriched terms is therefore consistent with the biological composition of the targeted panel and provides supportive, hypothesis-generating context for the experimentally observed transcriptional changes (Figure 8).

## 4. Discussion

The present study supports three principal observations. First, propofol produced a concentration-dependent reduction in the MTT signal in MIA PaCa-2 cells. Second, Annexin V-FITC/7-AAD analysis demonstrated an increase in the apoptotic fraction with minimal necrosis. Third, propofol exposure was associated with a selective transcriptional pattern characterised by decreased WNT5B, FZD5, and WNT11 and increased GSK3B expression. Importantly, these transcript-level findings do not establish direct activation or inhibition of Wnt–Frizzled signalling because pathway activity, protein abundance, phosphorylation state, and transcriptional reporter output were not measured.

The apparent difference between the MTT-derived IC_50_ and the viable-cell fraction determined by flow cytometry should not be interpreted as a contradiction between equivalent measurements. The MTT assay reflects cellular tetrazolium-reducing metabolic activity, whereas Annexin V/7-AAD analysis identifies phosphatidylserine exposure and loss of membrane integrity. Accordingly, an MTT-derived IC_50_ of 7.6 µM indicates an approximately 50% reduction in the normalised MTT signal and does not imply that 50% of the cells were dead. A cell population may therefore exhibit substantially reduced metabolic activity while a larger proportion of cells remains Annexin V−/7-AAD− at the same time point. Propofol has also been reported to affect mitochondrial function, providing an additional reason to avoid interpreting the MTT signal as a direct measure of cell number or lethality. Confirmation using an orthogonal non-metabolic viability assay would further strengthen interpretation of this finding.

The Wnt-related transcriptional changes should likewise be interpreted cautiously. WNT5B, WNT11, and FZD5 are positioned predominantly at the ligand–receptor level, whereas GSK3B is an intracellular kinase with context-dependent functions. Although increased GSK3B transcript abundance could be compatible with enhanced β-catenin turnover in some settings, GSK3B activity is importantly regulated through phosphorylation, including inhibitory Ser9 phosphorylation [13,14]. Therefore, the observed 2.1-fold increase in GSK3B mRNA cannot be directly translated into increased kinase activity or reduced canonical Wnt signalling. This distinction is particularly relevant because higher GSK3B transcript expression was associated with poorer survival in the exploratory patient dataset, whereas a previous protein-level pancreatic-cancer cohort reported a different prognostic relationship [15]. These divergent observations reinforce the need to distinguish transcript abundance from protein activity and clinical function.

The concurrent decreases in WNT11, WNT5B, and FZD5 may suggest that propofol exposure preferentially affects selected upstream Wnt-associated components rather than producing a pathway-wide transcriptional shutdown. Wnt signalling in PDAC is heterogeneous and involves both canonical and non-canonical ligand dependencies [5,10,11], while WNT11 has been associated with invasion-related non-canonical signalling in other tumour contexts [16]. However, the present study did not directly assess β-catenin activity, migration, invasion, or pathway rescue. Protein-level measurements of total and phosphorylated GSK3B, active β-catenin and downstream targets, β-catenin/TCF reporter assays, and functional migration and invasion experiments would therefore be appropriate next steps.

The complementary bioinformatic analyses provide broader biological context for the four transcripts prioritised from the in vitro findings. TCGA-PAAD analyses showed altered expression patterns of GSK3B, WNT5B, WNT11, and FZD5 in pancreatic tumours, while survival analyses demonstrated gene-specific rather than uniform prognostic associations. Genomic alterations involving these genes were generally infrequent, although copy-number changes were associated with differences in transcript abundance in selected cases. GSK3B expression was additionally associated with estimated infiltration of several immune-cell populations and showed a comparatively stronger negative gene-effect profile in DepMap than WNT5B, WNT11, and FZD5, although the observed dependency scores did not consistently reach the range associated with common-essential genes. Exploratory KEGG analysis provided pathway-level context, whereas DGIdb querying identified previously reported pharmacological relationships involving selected pathway-associated genes. These analyses are complementary and hypothesis-generating; none directly model propofol exposure or independently confirm a propofol-mediated mechanism.

The low-micromolar MTT-derived IC_50_ observed in the present study is lower than concentrations used in several previous experimental studies of propofol in cancer cells [17,18,19,20]. Importantly, the clinical, genomic, immune, dependency, pathway-enrichment, and pharmacological findings were not uniformly concordant with the direction of the in vitro transcriptional changes. Rather than weakening the experimental observations, this heterogeneity highlights the context-dependent biology of Wnt-associated genes and argues against a simple interpretation in which propofol uniformly suppresses an oncogenic Wnt pathway [21,22]. The integrated findings therefore identify a biologically relevant transcriptional pattern that warrants functional investigation without establishing causality between the observed transcript changes and propofol-induced apoptosis.

Nevertheless, this observation should not be extrapolated directly to clinical anaesthesia. The present experimental exposure was continuous for 24 h, whereas perioperative pharmacokinetics, free drug concentrations, tissue exposure, and the tumour microenvironment differ substantially in patients. Clinical studies comparing propofol-based and volatile anaesthesia have also produced mixed oncological findings [23,24,25,26,27]. The current study should therefore be regarded as an in vitro, hypothesis-generating investigation rather than evidence supporting therapeutic repositioning or a clinical oncological benefit of propofol.

## 5. Limitations

Several limitations should be considered when interpreting the present findings. First, the experimental work was conducted using only the MIA PaCa-2 pancreatic cancer cell line. Although this model is widely used in PDAC research, it cannot represent the molecular and phenotypic heterogeneity of pancreatic ductal adenocarcinoma. No additional PDAC cell line was included for independent validation, and no non-malignant pancreatic epithelial model was examined. Consequently, a tumour-selectivity index could not be calculated, and the observed effects should not be interpreted as tumour-selective. Future studies should validate these findings in at least one additional PDAC cell line with a contrasting molecular phenotype and in a non-malignant pancreatic epithelial model.

Second, the molecular analysis was restricted to a targeted 18-gene RT-qPCR panel. Changes in transcript abundance alone do not establish functional activation or inhibition of Wnt–Frizzled signalling. No protein-level measurements, phosphorylation-state analyses, pathway-reporter assays, rescue experiments, or loss- or gain-of-function approaches were performed. Furthermore, the commercial panel was originally developed for a different tumour context and therefore cannot provide an unbiased assessment of pathway-wide transcriptional responses. The molecular findings should consequently be interpreted as targeted transcript-level observations rather than definitive evidence of a specific Wnt–Frizzled mechanism.

Third, MTT was used as the sole metabolic viability assay. The difference between the MTT-derived response and the Annexin V-FITC/7-AAD viable-cell fraction at 7.6 µM propofol illustrates that these assays measure distinct biological properties and should not be interpreted interchangeably. Additional orthogonal approaches, including direct cell counting, membrane-integrity assays, ATP-based measurements, or clonogenic recovery assays, would help distinguish metabolic suppression from irreversible cell loss. In addition, exposure was limited to 24 h and no washout or recovery condition was included; therefore, the persistence and reversibility of the observed effects remain unknown.

The complementary bioinformatic analyses also have inherent limitations. TCGA-PAAD and other publicly available datasets consist of patient tumours with heterogeneous clinical backgrounds and contain no information linking the analysed molecular patterns specifically to propofol exposure. Associations among transcript abundance, genomic alterations, immune-cell infiltration estimates, and survival are therefore observational and cannot establish causality. Immune-infiltration results represent computational estimates rather than direct measurements of tumour immune composition. Similarly, DepMap dependency scores are derived from heterogeneous cancer cell-line models and cannot be assumed to reproduce the response of MIA PaCa-2 cells to propofol. KEGG enrichment was based on a targeted gene set and therefore should not be interpreted as unbiased pathway discovery, while DGIdb associations represent previously reported pharmacological relationships rather than experimentally validated drug combinations in the present model. These analyses should therefore be regarded as contextual and hypothesis-generating rather than confirmatory.

Finally, the study was conducted entirely in vitro. The public patient datasets do not contain information on propofol exposure, and the bioinformatic associations cannot be interpreted as evidence that propofol alters patient outcomes. The present findings also do not establish that comparable transcriptional or apoptotic responses occur at clinically achieved propofol exposures during anaesthesia or that such responses influence tumour recurrence, progression, or survival. Further mechanistic studies, multi-cell-line validation, non-malignant controls, and ultimately in vivo investigations are required before translational conclusions can be drawn.

## 6. Conclusions

Propofol exposure reduced MTT-derived metabolic activity and increased apoptosis in MIA PaCa-2 pancreatic cancer cells under the tested in vitro conditions. At the transcriptional level, treatment was associated with decreased WNT5B, WNT11, and FZD5 expression and increased GSK3B expression, indicating a selective pattern of changes among Wnt–Frizzled-related transcripts. These transcript-level alterations do not, however, establish direct functional modulation of Wnt–Frizzled signalling or demonstrate that this pathway mediates the observed apoptotic response.

The complementary bioinformatic analyses showed that the experimentally prioritised transcripts exhibit heterogeneous expression and genomic, prognostic, immune, dependency, pathway, and pharmacological associations in cancer-related datasets. These findings provide broader biological context for the in vitro observations but do not establish that propofol produces equivalent molecular effects in patient tumours or that the identified associations mediate its cellular effects.

Overall, the combined experimental and computational findings support further investigation of GSK3B, WNT5B, WNT11, and FZD5 as candidate components of the cellular response associated with propofol exposure. Validation in additional PDAC and non-malignant pancreatic cell models, together with protein-, phosphorylation-, and pathway-level functional analyses and ultimately in vivo studies, will be required to establish the mechanistic and translational significance of these observations.

## Figures and Tables

**Table 1:** Quantitative Annexin V-FITC/7-AAD results across independent biological experiments.

Cell Population	Vehicle Control, Mean ± SD (%)	Propofol 7.6 µM, Mean ± SD (%)	*p* Value
Viable cells	99.30 ± 1.057	82.80 ± 0.841	*p* < 0.05
Early apoptotic cells	0.65 ± 0.09	14.10 ± 1.26	*p* < 0.001
Late apoptotic cells	0.02 ± 0.001	2.94 ± 0.53	*p* < 0.001
Necrotic cells	0.00	0.16 ± 0.07	*p* < 0.05
Total apoptotic cells	0.67 ± 0.11	17.04 ± 2.40	*p* < 0.001

**Table 2:** RT-qPCR transcript changes after 24 h propofol exposure.

Gene	Fold Change (2^−ΔΔCq^)	Direction	Raw *p* Value	BH-Adjusted q Value	Significant After BH-FDR
*APC*	0.91	—	*p* > 0.05	q > 0.05	No
*APC2*	1.27	—	*p* > 0.05	q > 0.05	No
*CD151*	1.73	—	*p* > 0.05	q > 0.05	No
*CDH1*	0.85	—	*p* > 0.05	q > 0.05	No
*CSNK1G2*	1.62	—	*p* > 0.05	q > 0.05	No
*CSNK1G3*	1.51	—	*p* > 0.05	q > 0.05	No
*EIF4E*	1.89	—	*p* > 0.05	q > 0.05	No
*FAT2*	1.68	—	*p* > 0.05	q > 0.05	No
*FZD5*	0.36	Decreased	*p* < 0.001	0.001	Yes
*FZD6*	0.54	—	*p* > 0.05	q > 0.05	No
*GSK3B*	2.10	Increased	*p* < 0.05	0.041	Yes
*HHATL*	1.82	—	*p* > 0.05	q > 0.05	No
*LRP2*	1.37	—	*p* > 0.05	q > 0.05	No
*MC1R*	1.43	—	*p* > 0.05	q > 0.05	No
*RAB23*	1.90	—	*p* > 0.05	q > 0.05	No
*TCF7L1*	1.07	—	*p* > 0.05	q > 0.05	No
*WNT11*	0.42	Decreased	*p* < 0.05	0.027	Yes
*WNT5B*	0.25	Decreased	*p* < 0.001	0.001	Yes

Note: RT-qPCR statistical inference was performed using ΔCq values. Multiple-testing correction was applied across the complete 18-transcript family using the Benjamini–Hochberg false discovery rate procedure (Q = 5%). Raw *p* values are presented using the original reporting thresholds. Statistical significance after multiple-testing correction was defined as q < 0.05. FZD5, GSK3B, WNT11, and WNT5B remained statistically significant after BH-FDR correction, whereas the remaining transcripts did not (q > 0.05).

**Table 3:** Exploratory drug–gene interactions identified through DGIdb for selected pathway-associated genes. These associations are presented for pharmacological contextualisation only and do not imply a propofol-mediated mechanism or therapeutic combination.

Gene	Drug Name	Interaction Type	Drug Status	Therapeutic Indication
*GSK3B*	Lithium	Inhibitor	FDA-approved	Bipolar disorder, oncology
*APC*	Aspirin	Modulator	FDA-approved	Cardiovascular disease, cancer
*CSNK1G2*	PF-670462	Inhibitor	Experimental	Circadian rhythm disorders
*FZD5*	Vantictumab	Antagonist	Clinical trial	Cancer
*WNT11*	LGK974	Inhibitor	Clinical trial	Cancer

## Data Availability

The public survival data examined in this study are accessible through KM-Plotter. Experimental data generated in this study are available from the corresponding author on reasonable request.
